# Biogeochemical Cycling by a Low-Diversity Microbial Community in Deep Groundwater

**DOI:** 10.3389/fmicb.2018.02129

**Published:** 2018-09-07

**Authors:** Emma Bell, Tiina Lamminmäki, Johannes Alneberg, Anders F. Andersson, Chen Qian, Weili Xiong, Robert L. Hettich, Louise Balmer, Manon Frutschi, Guillaume Sommer, Rizlan Bernier-Latmani

**Affiliations:** ^1^Environmental Microbiology Laboratory, Environmental Engineering Institute, School of Architecture, Civil and Environmental Engineering, École Polytechnique Fédérale de Lausanne, Lausanne, Switzerland; ^2^Posiva Oy, Eurajoki, Finland; ^3^Science for Life Laboratory, School of Engineering Sciences in Chemistry, Biotechnology and Health, Department of Gene Technology, KTH Royal Institute of Technology, Stockholm, Sweden; ^4^Chemical Sciences Division, Oak Ridge National Laboratory, Oak Ridge, TN, United States

**Keywords:** subsurface, sulphate reducing bacteria, sulphide, metabolism, metagenomics, metaproteomics

## Abstract

Olkiluoto, an island on the south-west coast of Finland, will host a deep geological repository for the storage of spent nuclear fuel. Microbially induced corrosion from the generation of sulphide is therefore a concern as it could potentially compromise the longevity of the copper waste canisters. Groundwater at Olkiluoto is geochemically stratified with depth and elevated concentrations of sulphide are observed when sulphate-rich and methane-rich groundwaters mix. Particularly high sulphide is observed in methane-rich groundwater from a fracture at 530.6 mbsl, where mixing with sulphate-rich groundwater occurred as the result of an open drill hole connecting two different fractures at different depths. To determine the electron donors fuelling sulphidogenesis, we combined geochemical, isotopic, metagenomic and metaproteomic analyses. This revealed a low diversity microbial community fuelled by hydrogen and organic carbon. Sulphur and carbon isotopes of sulphate and dissolved inorganic carbon, respectively, confirmed that sulphate reduction was ongoing and that CO_2_ came from the degradation of organic matter. The results demonstrate the impact of introducing sulphate to a methane-rich groundwater with limited electron acceptors and provide insight into extant metabolisms in the terrestrial subsurface.

## Introduction

The terrestrial deep subsurface hosts diverse microbial communities capable of utilising a range of electron acceptors including nitrate, manganese, iron, sulphate, and carbon dioxide (Pedersen et al., 2008; Hallbeck and Pedersen, 2012; Bagnoud et al., 2016; Ino et al., 2016; Lau et al., 2016; Wu et al., 2016). Geogenic gases, such as hydrogen and methane, that migrate from deep crustal layers into shallower aquifers can serve as electron donors and sources of energy for the microorganisms (Pedersen, 2014). Hydrogen is a major fuel for chemolithoautotrophic populations that catalyse metal and sulphur transformations (Pedersen, 1997; Hernsdorf et al., 2017; Wu et al., 2017). Methane can be utilised by anaerobic methanotrophic archaea (ANME) which, either independently or with a microbial partner, couple the anaerobic oxidation of methane (AOM) to the reduction of sulphate (Boetius et al., 2000), nitrate (Haroon et al., 2013), iron or manganese (Beal et al., 2009; Ettwig et al., 2016).

The granitic subsurface of the Fennoscandian Shield underlying much of Sweden and Finland has been extensively studied, motivated largely by the construction of deep geological repositories for the storage of spent nuclear fuel. These studies demonstrate taxonomically and functionally diverse microbial populations at various locations and depths (Pedersen, 2013; Nyyssönen et al., 2014; Hubalek et al., 2016; Wu et al., 2016). Between 10^3^ and 10^7^ cells/ml^−1^ have been reported (Pedersen, 1997; Pedersen et al., 2008) and microorganisms were detected as deep as has been explored (>2,000 m depth) (Nyyssönen et al., 2014; Purkamo et al., 2016). Microbial turnover rates are expected to be slow in these oligotrophic subsurface environments; nevertheless, adenosine triphosphate (ATP) assays (Eydal and Pedersen, 2007), RNA gene surveys (Bomberg et al., 2015), and enrichment culturing (Rajala et al., 2015; Purkamo et al., 2017) indicate that microorganisms from the subsurface Fennoscandian Shield are both viable and active.

**FIGURE 1:**
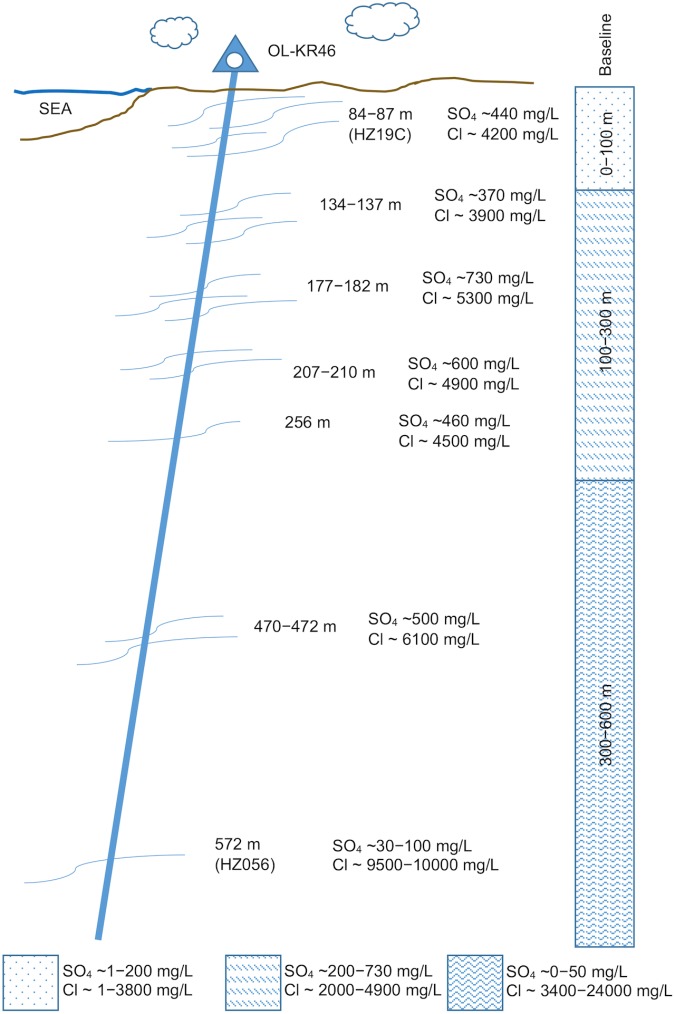
Drillhole OL-KR46, Olkiluoto, Finland. Reduced salinity and elevated sulphate concentrations are observed at depth in OL-KR46 due to down-hole mixing. Comparative baseline sulphate and chloride concentrations at Olkiluoto are indicated by the thatched boxes (Posiva, 2013). Depths indicate drillhole length, the fracture at 572 m corresponds to the studied fracture at 530.6 mbsl. HZ = modelled hydrological zones at Olkiluoto (Vaittinen et al., 2011).

The island of Olkiluoto has been selected as the site for a deep geological repository in Finland. The groundwater at Olkiluoto varies geochemically with depth, and can be broadly categorised into three water types; bicarbonate-rich, sulphate-rich and methane-rich (Posiva, 2013). Bicarbonate-rich groundwater is found at shallower depths (<100 m) and is fresh to brackish. Salinity increases with depth, and brackish sulphate-rich groundwater is found between ∼100 and 300 m depth (**Figure 1**). Below 300 m, sulphate is depleted and groundwater is methane-rich and saline. Where sulphate-rich and methane-rich groundwaters meet (250-350 m depth) elevated concentrations of sulphide have been observed (Posiva, 2013). In this mixing zone, most probable number (MPN) assays and 16S rRNA gene amplicon sequencing show an increased abundance and diversity of sulphate-reducing bacteria (SRB) (Pedersen, 2014). The elevated concentration of sulphide and the greater abundance of SRB suggest that SRB are active in the mixing zone. Also, sulphate and methane consumption was observed in experiments where flow cells were circulated with groundwater from the mixing zone and amended with hydrogen and/or methane (Pedersen, 2013). It was suggested that sulphate reduction may be coupled to methane oxidation, but the abundance of microorganisms known to perform AOM was found to be limited. In a separate study, 16S rRNA gene amplicons and methyl-coenzyme M reductase (*mcrA*) gene transcripts from putative AOM lineages were identified in the mixing zone (Bomberg et al., 2015). The evidence implicates methane as a possible electron donor for sulphate reduction in the mixing zone at Olkiluoto, but the process has not been clearly constrained.

The groundwater studied here also represents a mixing of sulphate-rich and methane-rich groundwater, but an artificial one, where mixing occurred as the result of down-drillhole flow of shallower sulphate-rich groundwater into deeper methane-rich groundwater when the hole was drilled (**Figure 1**). Though the fracture has since been isolated with packers and further mixing is precluded, sulphide concentrations are higher (∼1.5 mM) than those reported in the natural sulphate-methane mixing zone (∼0.5 mM) (Posiva, 2013). It is not clear whether methane oxidation could be contributing to the observed sulphide in this groundwater, or if sulphate reduction is coupled to an alternative electron donor.

The production of sulphide is problematic at this site as sulphide can corrode copper which will be used as the outermost canister material to store spent nuclear fuel. The planned repository will be at 400–450 m depth, below the sulphate-methane mixing zone, but artificial mixing could result from disturbances caused by repository construction. To explore the extant metabolisms and constrain the electron donor(s) fuelling sulphidogenesis in this case, we used metaproteogenomics to construct a metabolic model of methane-rich groundwater from a fracture at 530.6 m below sea level (mbsl). We explored the genes present within the microbial community to ascertain the functional potential, and also the protein complement as an indication of which metabolisms were active at the time of sampling. We further constructed metagenome assembled genomes (MAGs) to assign active metabolic function of specific taxonomic lineages.

## Materials and Methods

### Sample Site

The island of Olkiluoto, on the south-west coast of Finland, has been selected as the location for a deep geological repository. Groundwater was collected from a drillhole named OL-KR46 (61°14′31″N, 21°29′23″E) which was drilled in 2007 (**Figure 1**). In 2013, a low transmissivity fracture at 530.6 mbsl was isolated with double packers (packer interval 528.7-531.5 mbsl). During the 6-year interval prior to packering, the drillhole was left open, allowing the down-hole flow of sulphate-rich groundwater to deeper, methane-rich saline groundwater. Since the fracture was isolated, the concentration of sulphate has decreased from ∼4 mM to 0.6 mM and the concentration of chloride has increased from 7,440 mg/L to 10,300 mg/L (**Supplementary Table S1**) indicating that the fracture is slowly recovering from the influx of sulphate-rich brackish groundwater. Microbiological samples for this study were taken between March and November 2016. Prior to the first microbiological sampling, the fracture was pumped for 3 months. Geochemical parameters [pH, EC, dissolved O_2_ (DO), oxidation-reduction potential (ORP)] were monitored throughout the sampling period to ensure water was representative of the isolated fracture. In July 2016, during the sampling period, a packer pressure drop resulted in contamination of the fracture water with shallower sulphate-rich groundwater. The packer leak was repaired and pumping continued but microbiological samples were not collected in July and sampling resumed in August.

### Geochemical Analyses

#### Sulphate and Thiosulphate

Groundwater collected for the determination of sulphate and thiosulphate was filtered (0.22 μm pore size) into sterile 50 mL Falcon tubes and stored at 4°C. Sulphate and thiosulphate were measured by ion chromatography using a Dionex Integrion HPIC system with an IonPac AS18 analytical column. The flow rate was set to 0.25 mL/min and the eluent was KOH (40 mM).

#### Sulphide

Sulphide was fixed in solution by filtering (0.22 μm pore size) groundwater into sterile 15 mL Falcon tubes containing a zinc acetate solution (final concentration of 1%). Samples were stored at −20°C. Sulphide was measured spectrophotometrically on a Shimadzu UV-2501PC using the Cline assay (Cline, 1969).

#### Organic Compounds

Groundwater was filtered (0.22 μm pore size) into a sealed sterile 10 mL Balch tube by inserting a needle through the butyl rubber stopper. Samples were collected into sealed tubes to prevent loss of volatile compounds. Samples were stored at 4°C. Organic acids (acetate, lactate, propionate, butyrate) and glucose were measured on an Agilent 1290 Infinity LC (liquid chromatography) System fitted with an Agilent Hi-Plex H column with RI detection (Agilent, Santa Clara, CA, United States). Alcohols (ethanol, methanol, propanol, and 2-butanol) and acetone were measured on a Varian CP-3800 gas chromatograph (Agilent, Santa Clara, CA, United States) with 1-butanol as an internal standard.

#### Dissolved Gases (Methane and Hydrogen)

Using a needle, ∼10 mL of groundwater was collected into a sealed N_2_-filled 30 mL Wheaton glass serum bottle containing the biocide NaN_3_. Serum bottles were stored inverted, to minimise gas loss, at room temperature. The gas phase was sampled using a gas-tight syringe and H_2_ and CH_4_ measured on a Varian 450-GC (Agilent, Santa Clara, CA, United States) with a flame ionisation (FID) and thermionic specific (TSD) detector. Dissolved gas concentrations were calculated as described previously (Bagnoud et al., 2016).

#### Total and Dissolved Organic Carbon (TOC and DOC)

For TOC, groundwater was collected into sterile 50 mL Falcon tubes and stored at −20°C. For DOC, groundwater was filtered (0.22 μm pore size) into sterile 50 mL Falcon tubes and stored at 4°C. TOC and DOC were measured on a Shimadzu TOC-V.

#### Isotopic Composition of Sulphate

Sulphide in groundwater was fixed with a zinc acetate solution (final concentration 1%). The zinc sulphide precipitate was removed by centrifugation at 4,500 rcf for 10 min and the pellet was discarded. The supernatant was used for sulphur isotopic measurement of sulphate. The supernatant was acidified to ∼pH 3 with 1 M HCl and heated to 90°C in a water bath. Sulphate was precipitated as BaSO_4_ by the addition of BaCl_2_ (10% solution). Samples were left to cool overnight then centrifuged at 4,500 rcf for 10 min. The supernatant was discarded and the BaSO_4_ pellet air-dried. The ^32^S/^34^S isotope ratio of sulphate (δ^34^S_SO4_ ‰ VCDT) was measured at the Stable Isotope Laboratory (Faculty of Geosciences and Environment, Université de Lausanne, Lausanne, Switzerland). The S isotope composition was measured with a He carrier gas and a Carlo Erba (CE 1100) elemental analyser linked to a Thermo Fisher Delta V mass spectrometer. Samples were reacted at 1,050°C in a stream of He-carrier gas spiked with oxygen gas. External reproducibility of standards was <0.15‰ and samples were calibrated against IAEA standards S1 and S3 (Ag_2_S) and NBS-127 (BaSO_4_) with accepted values of −0.3, −32.1, and 20.3‰, respectively.

#### Isotopic Composition of Carbon (Dissolved Inorganic Carbon and Methane)

For analysis of the ^12^C/^13^C ratio of dissolved inorganic carbon (DIC), 30 mL glass serum bottles were rinsed three times in filtered MilliQ water and sealed with a butyl rubber stopper and crimped. Filtered (0.22 μm pore size) groundwater was collected into the serum bottles through a needle inserted into the butyl rubber stopper. Serum bottles were filled so that there was no headspace and stored at 4°C. The ^12^C/^13^C ratio of DIC (δ^13^C_DIC_ ‰ VPBD) was measured at the Stable Isotope Laboratory (Faculty of Geosciences and Environment, Université de Lausanne, Lausanne, Switzerland) with a Thermo Finnigan Delta Plus XL IRMS equipped with a GasBench II for analyses of carbonates. The C and O isotope composition of carbonates were measured with a GasBench II connected to a Finnigan MAT DeltaPlus XL mass spectrometer, using a He-carrier gas system according to methods adapted from Spötl and Vennemann (2003). Between 0.2 and 1.2 mL of water was injected into sample vials containing six drops of orthophosphoric acid that were flushed with He prior to injection. After 1 h of reaction and several minutes of agitation, the sample vials were inserted into the GasBench II at room temperature for analyses. Solid carbonate in-house standards were reacted with the same acid at 70°C for 1 h but their CO_2_ was also extracted at room temperature. Samples were normalised using an in-house standard calibrated against δ^13^C values of NBS-19 (+1.95‰, relative to VPDB). External reproducibility for the analyses estimated from replicate analyses of the in-house standard reacted at 110°C (*n* = 6) was ±0.04‰ for δ^13^C. The signal area of mass 44 of the samples was also used to calculate the concentration of DIC in solution. The precision of this method is 5% based on reproducibility of standard samples.

For analysis of the ^12^C/^13^C ratio of methane (δ^13^C_CH4_ ‰ VPBD), sealed serum bottles containing the biocide NaN_3_ were filled with groundwater so that there was no headspace. A known sample volume was extracted with a syringe flushed with synthetic air, the syringe was then shaken for ∼1 min to create a headspace. The ^12^C/^13^C ratio of methane in the headspace was measured using a Picarro G2201-I Analyser.

### Microbiological Analyses

#### Cell Enumeration

Groundwater was collected into a sterile Falcon tube containing particle-free paraformaldehyde (final concentration of 1.6%). Fixed samples were stored at 4°C. For cell enumeration, 10 mL of the fixed sample was filtered through a black polycarbonate filter membrane (Merck Millipore, Darmstadt, Germany) and washed three times with PBS (8 g/L NaCl, 0.2 g/L KCl, 1.4 g/l Na_2_HPO_4_, 0.24 g/l KH_2_PO_4_, pH 7.4). The filter membrane was stained with SYBR Green (Thermo Fisher Scientific, Inc.) for 20 min. Cells were then imaged on an epi-fluorescent Nikon Eclipse E800 microscope at 1,000× magnification. Cell numbers were calculated from 25 fields of view.

#### Groundwater Filtration

To collect biomass for DNA and protein analysis, groundwater was pumped directly into a chilled sterile Nalgene filtration unit fitted with a 0.22 μm pore size Isopore polycarbonate membrane (Merck Millipore, Darmstadt, Germany) and connected to a vacuum pump. Up to 10 L of groundwater was filtered to obtain a sufficient DNA yield for metagenomic sequencing (25 ng total DNA) and 7 L was filtered for metaproteomic analysis. Filters were removed aseptically and stored in 1.5 mL sterile screwcap tubes. Filters collected for DNA were preserved in 750 μL LifeGuard Soil Preservation Solution (MoBio, Carlsbad, CA, United States). Filters collected for protein were flash-frozen in a dry ice and ethanol mixture. All filter tubes (for DNA and protein extraction) were stored on dry ice on site. Filters for DNA were subsequently stored at −20°C and filters for protein were stored at −80°C.

#### DNA Extraction

DNA was extracted from filters collected in March, May, June, August, September, and November using a phenol-chloroform method described previously (Bagnoud et al., 2016) with the following modifications; (1) filter pieces were subject to bead-beating (2 × 15 s) prior to incubation in lysozyme for 2 h at 37°C; (2) lysate was incubated in Proteinase K (200 μg/mL final concentration) for 2 h. DNA was quantified on a Qubit 3.0 Fluorometer using the Qubit dsDNA High Sensitivity Assay kit (Thermo Fisher Scientific, Inc.).

#### 16S rRNA Gene Amplicon Sequencing

Extracted DNA was used as a template for PCR amplification using primers 515F/806R that target the V4 region of the 16S rRNA gene (Caporaso et al., 2011). Libraries were generated on an Illumina MiSeq at either RTL Genomics, Lubbock, TX, United States, or the Lausanne Genomics Technologies Facility at Université de Lausanne (UNIL), Lausanne, Switzerland, with a 2 × 250 bp read configuration resulting in an ∼200 bp overlap. Sequence reads were merged and quality filtered with USEARCH v10 (Edgar, 2010). Operational taxonomic units (OTUs) were assigned and chimeras removed using the UNOISE2 algorithm (Edgar, 2016). Taxonomy was assigned to OTUs with RDP classifier (Cole et al., 2009) in QIIME (Caporaso et al., 2010) using the Silva 128 database (Quast et al., 2013). Rarefaction to the lowest number of sequences (*n* = 34,306) was used to normalise the sample count prior to analysis of the dataset. Raw 16S rRNA gene amplicon reads are available at the NCBI Sequence Read Archive (SRA) under BioProject accession PRJNA472445.

#### Metagenomic Sequencing and Construction of MAGs

Three metagenomes were generated from DNA extracted in March, June, and August. Metagenomic sequencing was performed at two facilities; the Joint Genome Institute (JGI), Walnut Creek, CA, United States and the Marine Biological Laboratory (MBL), Woods Hole, MA, United States. Briefly, DNA processed at the MBL was sheared into fragments (∼275 bp) prior to library preparation. The paired end library was constructed with the Ovation Ultralow DR Multiplex System 1-96 (Nugen Technologies, United States) and 15 cycles of PCR was used to enrich for the final library. After library amplification, size selection was performed with a Pippen Prep (Sage Science, United States). Sequencing was performed on an Illumina NextSeq platform with a 2 × 150 bp read configuration. DNA processed at the JGI was sheared into 300 bp fragments and libraries were prepared with the KAPA-Illumina library creation kit (KAPA biosystems) and 5–15 cycles of PCR was used to enrich for the final library. The libraries were quantified using the KAPA Biosystems next-generation sequencing library qPCR kit and run on a Roche LightCycler 480 real-time PCR instrument. Sequencing was performed on an Illumina HiSeq platform with a 2 × 150 bp read configuration. Sequencing data were deposited at the NCBI SRA under the BioProject accession numbers PRJNA404457, PRJNA404458, and PRJNA472439.

Metagenomic sequence reads from both sequencing facilities were pre-processed using FastUniq (Xu et al., 2012). A separate assembly was performed for each of the three samples using MegaHit (Li et al., 2015). Assembled contigs were quantified across all samples using Kallisto (Bray et al., 2016). Genes were identified using Prodigal (Hyatt et al., 2010) and annotated with KEGG Orthology (KO) assignments using GhostKOALA (Kanehisa et al., 2016), which assigns each query gene a functional annotation as well as a taxonomic category according to the best-hit gene. Ribosomal sequences were identified and taxonomically classified with Metaxa2 (Bengtsson-Palme et al., 2015). 16S rRNA gene sequences were also compared to the NCBI 16S ribosomal RNA database for bacteria and archaea using BLAST (Altschul et al., 1990).

Contigs were binned according to sequence composition and coverage using CONCOCT (Alneberg et al., 2014). Binning was performed on each sample separately, using the abundance profile over all samples. Bins were assessed for contamination and completeness using CheckM (Parks et al., 2015) which uses lineage specific marker genes to assess the bin quality. Bins that were considered high quality (>90% completeness, <5% contamination) or good quality (>75% completeness, <10% contamination) were considered to be a MAG and were retained for further analysis. MAGs were clustered into groups using based on nucleotide identity using MUMmer (Kurtz et al., 2004). MAGs from the same group are considered to represent the same taxonomic lineage from different sampling time points. Gene prediction and assignment of KO identifiers was performed on each MAG as described above. Hydrogenases were classified using HydDB (Søndergaard et al., 2016). KEGG Mapper (Kanehisa et al., 2012) and MetaCyc (Caspi et al., 2018) were used to reconstruct metabolic pathways.

#### Metaproteomic Analysis

Protein was extracted from filters collected in August at the Oak Ridge National Laboratory (Oak Ridge, TN, United States). Filters were placed into 1 mL lysis buffer containing 100 mM Tris-HCl, pH 8.0, 4% w/v SDS (sodium dodecyl sulphate), and 10 mM dithiothreitol (DTT), and then subjected to 15 min of boiling (Chourey et al., 2013). Samples were cooled at room temperature and the supernatant containing the crude protein extract was transferred to clean tubes. Proteins were precipitated by adding chilled 100% TCA to a final concentration of 20% v/v and stored at −80°C overnight. Protein pellets were washed with ice-cold acetone and further resuspended in 8 M urea, 100 mM Tris-HCl, pH 8.0 with the assistance of limited sonication. Denatured proteins were further reduced by DTT and alkylated by iodoacetamide (IAA). Sequencing grade trypsin (Promega, Madison, WI, United States) was employed in a sequential double-digestion to ensure complete proteolysis. Following digestion, the peptide solution was adjusted to 200 mM NaCl, 0.1% formic acid (FA) and filtered through a precleaned (with 100 mM Tris-HCl, pH 8.0 buffer) 10 kDa molecular weight cut off spin column filter (Vivaspin 2, GE Healthcare Life Sciences, Pittsburgh, PA, United States). The cleaned peptide samples were quantified by the BCA assay (Pierce Biotechnology, Waltham, MA, United States) and stored at −80°C until analysis by mass spectrometry (Qian et al., 2016). Proteolytic peptides (10 μg) were loaded onto a biphasic silica back-column that consists of reverse-phase (C18) resin (Aqua, Phenomenex) followed by strong cation exchange (SCX) resin (Luna, Phenomenex). The back-column was then coupled in-line with an in-house pulled, 12 cm reverse-phase packed analytical column (100 μm i.d.) interfaced with an LTQ-Orbitrap-Elite mass spectrometer (Thermo Scientific). Due to the limited amount of sample, peptides were analysed by a modified MudPIT (multidimensional protein identification technology) approach which consists of three salt pulses (10, 25, and 100% of 500 mM ammonium acetate). The LTQ-Orbitrap-Elite was operated in a data dependent mode with the same parameters described previously (Xiong et al., 2015). Three technical replicates were analysed.

A protein database was constructed from protein sequences predicted from the assembled genomes. All collected MS/MS spectra were searched against protein databases using Myrimatch v2.2 (Tabb et al., 2007). Peptides were identified and assembled into proteins using IDPicker v3.1 (Ma et al., 2009) with a minimum of two distinct peptides per protein and a false discovery rate (FDR) of <1% at the peptide level. Proteins were further clustered into protein groups post database searching if all proteins shared the same set of identified peptides. At least one additional peptide was required for the formation of a new protein group.

## Results

### Groundwater Characterisation

Groundwater from the drillhole OL-KR46 was collected from a fracture at 530.6 mbsl. The groundwater was temperate (12.6°C), saline (TDS 15.8 g/L), alkaline (pH 8.8), anoxic (<12 ppb dissolved O_2_) and reducing (ORP −525 mV). Total cell number was estimated to be 2 × 10^5^ cells/mL^−1^, which is within the range previously reported for Olkiluoto groundwater (Pedersen et al., 2008). The groundwater was characterised by low DIC (0–2 mg/L), high chloride (9.8 ± 0.8 g/L) and high methane (1.5 ± 0.4 mM) (**Table 1** and **Supplementary Table S1**). Sulphate was present (1.0 ± 0.3 mM) as a result of mixing with shallower sulphate-rich groundwater (see section “Sample Site”) and was greatest in August (**Table 1**) following an influx of shallower sulphate-rich groundwater. This influx of shallower groundwater was also evident by decreased salinity (**Supplementary Table S1**). Overall the sulphate concentration showed a general decreasing trend over the 9-month sampling period (**Table 1**). The sulphide concentration (1.3 ± 0.4 mM) is among the highest observed in Olkiluoto groundwater and thiosulphate is also relatively abundant (0.4 ± 0.2 mM). The stable isotopic composition of sulphate (δ^34^S_SO4_) was +27.26 ± 2.34‰ VCDT. The carbon stable isotopic ratio of methane (δ^13^C_CH4_) and DIC (δ^13^C_DIC_) was −32.20 ± 0.49‰ VPDB and −15.68 ± 1.37‰ VPDB, respectively. DOC was high (4.7 ± 0.7 mM) and is contributed to by the organic compounds acetate (1.5 ± 0.3 mM), acetone (8.1 ± 3.6 μM), ethanol (28.6 ± 16.7 μM), methanol (65.4 ± 22.7 μM) and 2-butanol (3.7 μM ± 1.3 μM). Hydrogen, formate, lactate, propionate, pyruvate, butyrate, and glucose were measured but not detected.

**Table 1:** Geochemical characteristics of groundwater from a fracture at 530.6 mbsl in drillhole OL-KR46.

	March	May	June	August	September	November
Sulphate (mM)^1^	1.13 ± 0.09	1.01 ± 0.21	0.96 ± 0.23	1.67 ± 0.02	0.78 ± 0.07	0.83 ± 0.07
Thiosulphate (mM)^1^	0.51 ± 0.14	0.33 ± 0.02	0.43 ± 0.11	0.57 ± 0.15	0.25 ± 0.01	0.18 ± 0.01
Sulphide (mM)^2^	1.51 ± 0.11	–	1.69 ± 0.35	0.92 ± 0.06	1.14 ± 0.26	1.12^∗^
∂^34^S_SO4_ ‰ VCDT^1^	30.43 ± 0.83	28.75 ± 0.23	25.06 ± 0.56	24.62 ± 0.11	27.45 ± 0.13	–
Methane (mM)^3^	1.71 ± 0.36	1.26 ± 0.18	1.63 ± 0.49	1.06 ± 0.28	1.69 ± 0.22	1.40 ± 0.05^4^
∂^13^C_CH4_ ‰ VPBD^1^	−32.56 ± 0.36	−31.67 ± 0.01	−31.66 ± 0.09	−32.16 ± 0.25	−32.65 ± 0.34	–32.69^∗^
DIC (mg/L)	2.1	1.7	<1.6	3.3	<1.6	<1.6
∂^13^C_DIC_ ‰ VPBD^1^	−14.52 ± 0.47	−16.85 ± 0.12	–	–	DIC too low	DIC too low
TOC (mg/L)^1^	59.68 ± 0.62	64.81 ± 0.33	65.40 ± 0.32	51.65 ± 2.81	52.50 ± 4.10	61.64^∗^
DOC (mg/L)^1^	62.71 ± 14.51	56.27 ± 0.26	56.77 ± 0.92	43.78 ± 0.91	59.40 ± 1.12	61.18^∗^
Acetate (mM)^1^	1.01 ± 0.47	1.48 ± 0.12	1.60 ± 0.00	1.63 ± 0.01	1.22 ± 0.59	–
Acetone (μM)^1^	13.09 ± 2.05	7.97 ± 0.72	7.65 ± 0.64	4.76 ± 1.45	7.22 ± 6.16	–
Ethanol (μM)^1^	32.18^∗^	33.16^∗^	25.52 ± 2.99	17.99 ± 1.55	52.33 ± 0.61	–
Methanol (μM)^1^	60.09 ± 9.80	84.54 ± 53.94	58.00 ± 1.94	52.04 ± 15.89	72.38 ± 5.33	–
2-Butanol (μM)^1^	3.40 ± 0.30	4.09 ± 0.08	3.78 ± 0.03	2.19 ± 0.09	5.10 ± 2.25	–

*DIC = dissolved inorganic carbon, TOC = total organic carbon, DOC = dissolved organic carbon. ^1^ = ± standard deviation (n = 2), ^2^ = ± standard deviation (n = 4), ^3^ = ± standard deviation (n = 6), ^4^ = ± standard deviation (n = 3), ^∗^ = (n = 1), = not measured.*

### Community Composition

**FIGURE 2:**
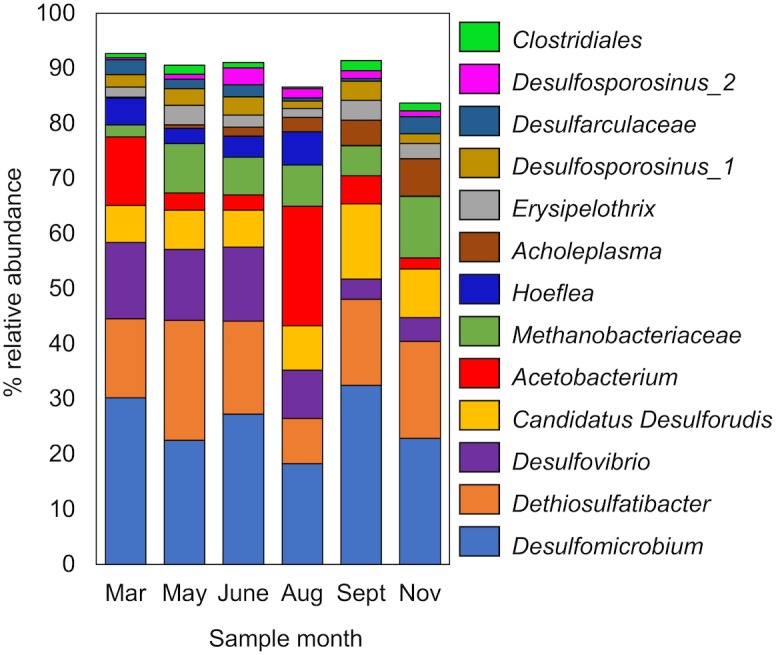
Abundant OTUs (>1% relative abundance) in 16S rRNA gene amplicon libraries from OL-KR46 methane-rich groundwater. OTUs were detected from the phyla; *Firmicutes* (*Dethiosulfatibacter*, Candidatus *Desulforudis*, *Acetobacterium*, *Erysipelothrix*, *Desulfosporosinus*, and *Clostridiales*), *Deltaproteobacteria* (*Desulfarculaceae*, *Desulfovibrio*, and *Desulfomicrobium*), *Tenericutes* (*Acholeplasma*), *Alphaproteobacteria* (*Hoeflea*), and *Euryarchaeota* (*Methanobacterium*). All OTUs are provided in **Supplementary Table S3**.

Six 16S rRNA gene amplicon libraries were generated from DNA extracted from groundwater samples collected over the 9-month sampling period. Analysis of 16S rRNA gene amplicons showed that the microbial population was predominantly bacterial (92.9 ± 2.8% relative abundance). *Firmicutes* (44.0 ± 3.1%) and *Proteobacteria* (42.4 ± 7.8%) were the most abundant bacterial phyla. All archaeal sequences belonged to the phylum *Euryarchaeota* (7.1 ± 3.1%). Microbial diversity was low (**Supplementary Figure S1**), with 13 abundant (≥1% average relative abundance) OTUs representing 83.7–92.7% of the total community (**Figure 2**). Lineages known to reduce sulphur compounds had the greatest relative abundance, accounting for up to 74% of the total microbial community. This included the OTUs *Desulfomicrobium*, *Dethiosulfatibacter*, *Desulfovibrio*, and *Desulfarculaceae* from the *Deltaproteobacteria*, and Candidatus *Desulforudis* and *Desulfosporosinus* from the *Firmicutes* (**Figure 2**). The *Desulfovibrio* OTU detected shared 100% sequence identity (250 nt) with *Pseudodesulfovibrio aespoeensis* (formerly *Desulfovibrio aespoeensis*), a hydrogenotrophic (hydrogen-oxidising) sulphate reducer isolated from 600 m depth at Äspö, which forms part of the Fennoscandian Shield in Sweden (Motamedi and Pedersen, 1998; Pedersen et al., 2014). Other abundant OTUs included a methanogen (*Methanobacteriaceae*), an acetogen (*Acetobacterium*) as well as putative nitrate-reducing (*Hoeflea*) and fermentative microorganisms (*Acholeplasma*, *Erysipelothrix*, and *Clostridiales*) (**Figure 2**).

### Metaproteogenomics and MAGs

**FIGURE 3:**
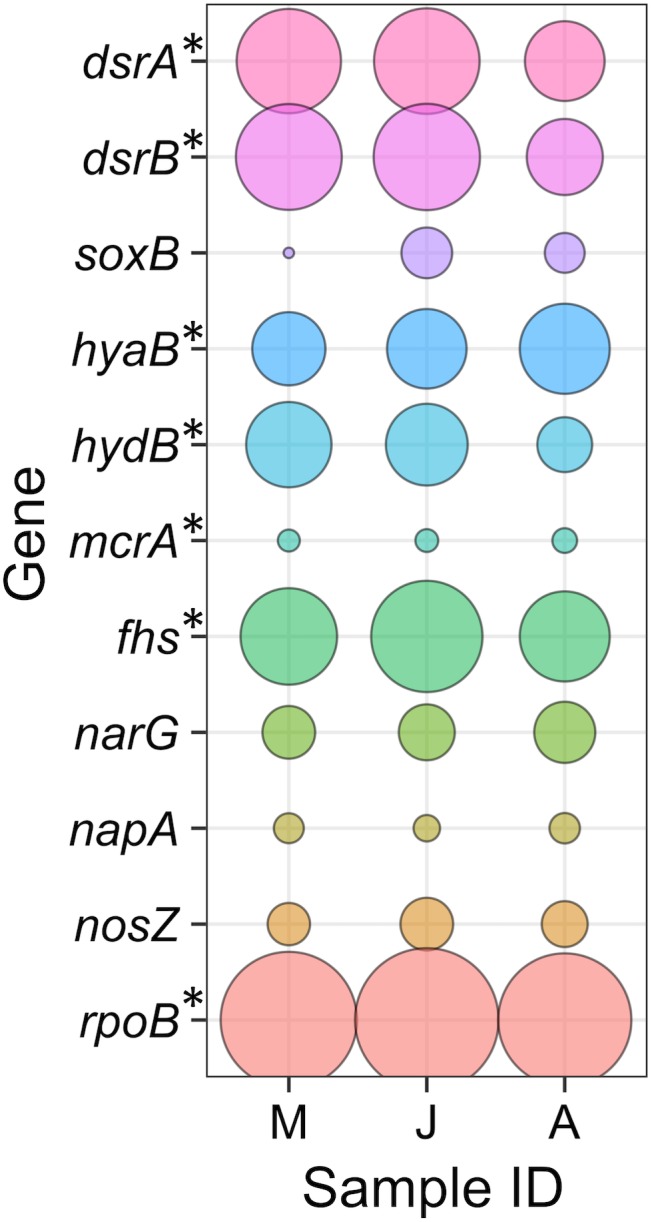
Genes from key metabolic pathways of interest. Gene abundances are shown relative to the beta subunit of the single-copy RNA polymerase (*rpoB*) present in all microorganisms. Genes with an asterisk (^∗^) were detected in the metaproteome. Gene descriptions are provided in the text. Sample IDs denote the sample months May (M), June (J), and August (A).

Three metagenomic libraries were generated from DNA extracted from groundwater samples taken in March, June, and August 2016 (**Supplementary Table S2**). Genes encoding key enzymes from metabolic pathways of interest were sought within the metagenome and metaproteome. This indicated that sulphate reduction, hydrogen oxidation and methanogenesis are active metabolisms and that there is the metabolic potential for nitrate reduction and denitrification (**Figure 3**). To determine which taxonomic lineages carried out these metabolisms, assembled contigs were binned according to their coverage and sequence composition (Alneberg et al., 2014). A total of 21 bins were retained for further analysis based on their level of completeness and potential contamination (**Table 2**). Out of the 21 bins retained, 13 had >90% completeness and <5% contamination and were considered to be high quality MAGs. Seven bins were considered as good quality MAGs with >75% completeness and <10% contamination. One lower quality bin (number 19) was retained (68% completeness and 0.7% contamination) as it had low contamination and shared the same taxonomy as a good quality MAG recovered from groundwater from another Olkiluoto drillhole (OL-KR13). The good quality MAG from OL-KR13 was therefore used to check for genes that were missing in the less complete OL-KR46 MAG.

**Table 2:** MAGs constructed from OL-KR46 metagenomes.

MAG group	Bin	Taxonomy	Marker lineage	Sample	Completeness/Contamination (%)		Strain heterogeneity	Genome size (Mbp)	Number of contigs	Mean abundance (% of *rpoB)*
1	1	*Hoeflea*	Rhizobiales	March	99.86	0.58	0.00	4.82	46	5.2
	2			June	99.86	0.78	0.00	5.28	95	11.1
	3			August	95.21	0.39	0.00	4.13	29	5.7
2	4	*Desulfomicrobium*	Delta-proteobacteria	March	97.02	0.20	0.00	4.32	71	12.3
	5			June	92.24	0.00	0.00	3.81	74	16.3
	6			August	97.00	0.00	0.00	3.85	45	8.2
3	7	*Desulfarculus*	Delta-proteobacteria	March	97.42	0.65	0.00	4.45	55	6.6
	8			June	96.13	1.29	0.00	4.35	93	4.5
	9			August	95.40	1.94	33.33	4.01	467	1.5
4	10	Candidatus *Desulforudis*	Firmicutes	March	95.54	3.03	0.00	2.05	289	1.8
	11			June	100.00	3.18	0.00	2.34	137	2.5
	12			August	100.00	3.18	0.00	2.25	68	4.6
5	13	*Hydrogenophaga*	Burkholderiales	August	89.15	4.51	46.15	3.25	779	1.2
6	14	*Methanobacterium*	Euryarchaeota	March	77.34	0.84	0.00	1.54	675	0.8
	15			June	100.00	1.60	0.00	2.17	121	1.1
	16			August	87.54	2.40	0.00	1.80	354	1.7
7	17	*Clostridiales*	Clostridiales	June	90.91	6.99	63.16	3.40	328	4.6
8	18	*Erysipelothrix*	Bacteria	June	85.26	8.81	41.67	1.90	437	1.1
9	19	*Acetobacterium*	Clostridiales	August	68.16	0.70	0.00	1.97	138	3.8
10	20	*Pseudodesulfovibrio*	Bacteria	March	96.49	10.53	5.00	3.59	73	29.9
	21			August	96.49	10.53	5.00	3.59	70	13.5

Clustering of the 21 OL-KR46 MAGs based on nucleotide identity resulted in 10 distinct groups of MAGs representing different taxonomies (**Table 2**). The 16S rRNA amplicon sequencing results (**Figure 2**) were compared to 16S rRNA sequences in individual MAGs (when present) and used for taxonomic assignment. A MAG from group 3 included an identical 16S rRNA gene sequence to the OTU *Desulfarculaceae*, resulting in the assignment of MAG group 3 to that family (**Table 2** and **Figure 2**). The longer 16S rRNA gene sequence (v1-v9) obtained from the MAG enabled further classification of this taxon to the genus *Desulfarculus*. The MAG from group 9 contained a complete 16S rRNA gene sequence identical to the OTU *Acetobacterium* (**Figure 2**). A 16S rRNA gene sequence recovered from a group 4 MAG was reassigned to group 8 based on the taxonomic annotation of other genes in the MAG. The 16S rRNA gene sequence assigned to group 8 was identical to the OTU *Erysipelothrix* (**Figure 2**). The remaining seven groups of MAGs did not contain a 16S rRNA gene sequence. These MAGs were classified according to the GhostKOALA taxonomic annotation as: *Desulfomicrobium*, Candidatus *Desulforudis*, *Pseudodesulfovibrio* (formerly *Desulfovibrio*), *Methanobacterium*, *Clostridiales*, *Hoeflea*, and *Hydrogenophaga* (**Table 2**). Except *Hydrogenophaga*, all of these taxonomies were identified as abundant OTUs by 16S rRNA gene amplicon sequencing (**Figure 2**). *Hydrogenophaga* was detected in 16S rRNA gene amplicon libraries in low relative abundance (0.0–0.9%) (**Supplementary Table S3**).

### Sulphate-Reducing Bacteria

Sulphate reduction was evident by the presence of the genes encoding the alpha and beta subunits of the dissimilatory sulphite reductase (*dsrA* and *dsrB*, EC:1.8.99.5) which catalyses the final step in sulphate reduction, reducing sulphite to sulphide. The *dsrAB* genes were abundant in metagenomic libraries (**Figure 3**), consistent with the abundance of SRB detected by 16S rRNA gene amplicon sequencing (**Figure 2**). Peptides from SRB DsrAB were also detected in the metaproteome, indicating that sulphate reduction is active. However, the *dsrAB* genes are not exclusive to sulphate reduction as the proteins they encode can operate in the reverse direction during sulphide oxidation (Loy et al., 2009). A small proportion (∼5%) of the *dsrAB* reads were related to the sulphide-oxidising *Hydrogenophaga*, but peptides from the corresponding proteins were not detected in the metaproteome.

**FIGURE 4:**
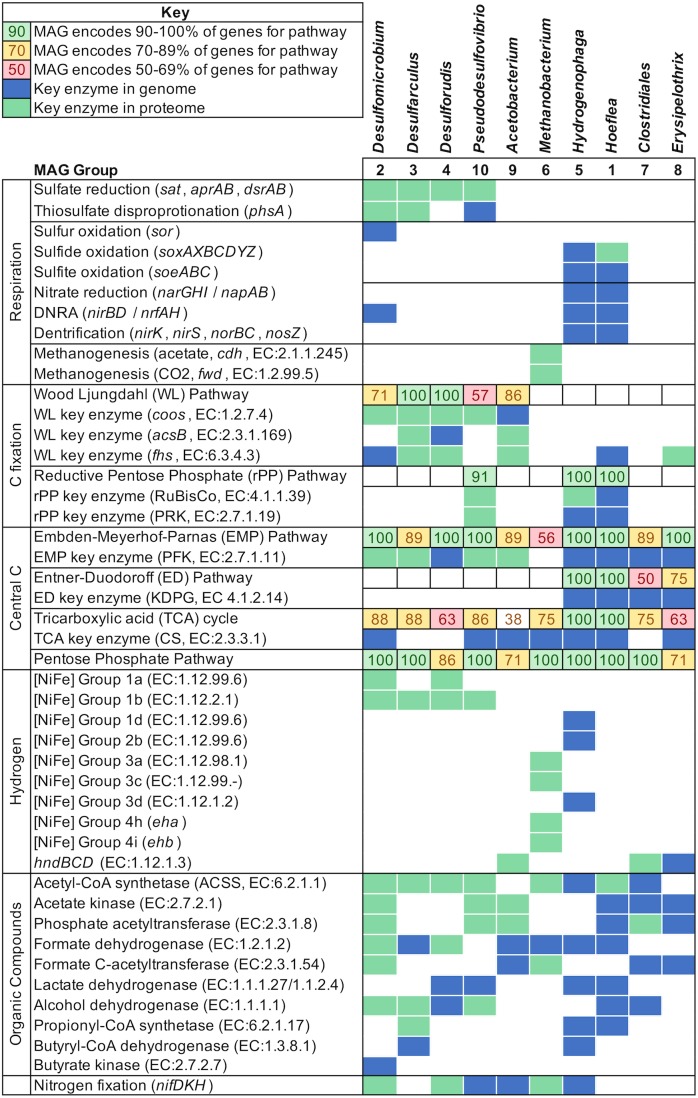
Key metabolic pathways identified in MAGs constructed from groundwater from drillhole OL-KR46. Only complete or near complete pathways are shown.

The MAGs *Desulfomicrobium*, *Desulforudis*, *Desulfarculus*, and *Pseudodesulfovibrio* harboured the complete pathway for dissimilatory sulphate reduction in their proteome (**Figure 4**). In addition, the *Desulfomicrobium* and *Pseudodesulfovibrio* MAGs encoded Qmo (quinone-interacting membrane-bound oxidoreductase) as a *sat-aprBA-qmoABC* gene cluster, which is present in the majority of, but not all, sulphate-reducing microorganisms (Pereira et al., 2011). In *Desulforudis* and *Desulfarculus, qmoC* was replaced with *hdrBC* genes that encode for subunits of heterodisulphide reductase. This gene arrangement is usually found in Gram-positive sulphate-reducing microorganisms (Grein et al., 2013), although *Desulfarculus* is reported to be Gram-negative (Sun et al., 2010). The proteomes of the *Deltaproteobacterial* MAGs *Desulfomicrobium*, *Desulfarculus* and *Pseudodesulfovibrio* also harboured a subunit of the protein thiosulphate reductase (PhsA), which catalyses the initial step in the disproportionation of thiosulphate and is relatively common among sulphate-reducing *Deltaproteobacteria* (Thorup et al., 2017). The *Desulfomicrobium* MAG additionally harbours a sulphur oxygenase reductase (*sor*) which typically encodes a protein that catalyses the oxygen-dependent disproportionation of elemental sulphur (Janosch et al., 2015). The *sor* gene has also been reported in the sulphate- and sulphur-reducing *Desulfomicrobium baculatum*, but the physiological role of this enzyme in strictly anaerobic bacteria is unclear (Veith et al., 2012). Evidence of the expression of the *sor* gene was not found in the proteome of *Desulfomicrobium*.

*Desulfomicrobium*, *Desulforudis*, and *Pseudodesulfovibrio* demonstrated evidence for expression of genes for nitrogen fixation in their proteome (*nifDKH*; EC:1.18.6.1) despite the nitrogenase conversion of N_2_ to ammonia being energetically costly. Ammonium transporters were identified in the genomes of all SRB MAGs but only *Desulforudis* expressed an ammonium transporter in its proteome. All SRB expressed carbon-monoxide dehydrogenase (*cooS*, EC:1.2.7.4), a key enzyme in the Wood–Ljungdahl (WL) pathway, in their proteome. Only *Desulfarculus* and *Desulforudis* had the complete WL pathway including a second key enzyme, acetyl-CoA synthase (*acsB*, EC:2.3.1.169) (**Figure 4**). The WL pathway was incomplete in the genomes of *Desulfomicrobium* and *Pseudodesulfovibrio* (**Figure 4**). *Pseudodesulfovibrio* harboured an alternative pathway for carbon fixation via the reductive pentose phosphate (rPP) pathway. Genes from the rPP pathway were found in the proteome of *Pseudodesulfovibrio*, including the key enzymes ribulose biphosphate carboxylase (RuBisCo, EC:4.1.1.39) and phosphoribulose kinase (PRK, EC:2.7.1.19) (**Figure 4**). The reverse tricarboxylic acid cycle (rTCA) was incomplete in all SRB MAGs.

All SRB MAGs included acetyl-CoA synthetase (ACSS, EC:6.2.1.1) in their proteome for the interconversion of acetate and acetyl-CoA (**Figure 4**). *Desulfomicrobium* and *Pseudodesulfovibrio* additionally had an alternative pathway for acetate metabolism via phosphate acetyltransferase (*pta*, EC:2.3.1.8). Enzymes for the oxidation of other small organic compounds were also detected in the genomes and proteomes of the SRB MAGs (**Figure 4**). In addition to small organic compounds, SRB possessed genes for glycolysis via the Embden–Meyerhof–Parnas (EMP) pathway and pyruvate oxidation (**Figure 4**). The pathway was partially identified in the proteome of *Desulfomicrobium*, Candidatus *Desulforudis* and *Pseudodesulfovibrio* suggesting their metabolism can also be fermentative.

Hydrogenotrophic respiration within the microbial community was evidenced by the presence of genes encoding the large subunits of hydrogenases *hyaB* (EC:1.12.99.6) and *hydB* (EC:1.12.2.1). Both hydrogenases are Group 1 respiratory H_2_-uptake [NiFe] hydrogenases. The genes encoding these hydrogenases were abundant in the metagenome and the associated proteins were detected in the metaproteome (**Figure 3**). All SRB MAGs expressed Group 1 H_2_-uptake [NiFe] hydrogenases (**Figure 4**).

### Homoacetogenic Bacteria

The enzyme formyl tetrahydrofolate synthetase (encoded by *fhs*, EC:6.3.4.3) catalyses the ATP-dependent activation of formate in the WL pathway which can be used to indicate acetogenesis (Lever, 2013). However, as indicated above, the pathway can also be found in SRB and acetoclastic methanogens and utilised in reverse, for acetate oxidation (Ragsdale and Pierce, 2008). The abundance of this gene in the metagenome (**Figure 3**) indicates that a large proportion of the microorganisms in the population are able to utilise the WL pathway (in either direction) and the presence of peptides of this gene in the metaproteome indicates microorganisms using this pathway are active. However, reliable interpretations of active homoacetogenesis are not possible with this gene alone.

However, a MAG related to *Acetobacterium* was identified and presumed to represent a homoacetogen. This MAG encoded a near-complete WL pathway and genes encoding key enzymes (*fhs* and *acsB*) were observed in its proteome (**Figure 4**). Subunits of the NADP-reducing hydrogenase (*hndBCD*; EC:1.12.1.3) were also abundant in the *Acetobacterium* proteome (**Figure 4**). Homoacetogens use the WL pathway as a mechanism for energy conservation and autotrophic carbon assimilation coupling hydrogen oxidation to carbon dioxide reduction (Ragsdale and Pierce, 2008). They can also produce acetate via fermentation of sugars. *Acetobacterium* encoded a near complete EMP pathway for fermentative acetate production. The EMP pathway was partially identified in the proteome, suggesting *Acetobacterium* can utilise both organic and inorganic carbon compounds to generate acetate.

### Methanogenic Archaea

The alpha subunit of methyl-coenzyme M reductase (encoded by *mcrA*, EC:2.8.4.1) catalyses both production of methane (methanogenesis) and the AOM. The *mcrA* gene had low relative abundance in the metagenome but peptides from this subunit were detected in the metaproteome (**Figure 3**). The *mcrA* genes identified were related to the methanogen *Methanomicrobium* suggesting that the *mcrA* enzyme operates in the methane producing direction. Methanogenic archaea conserve energy by the conversion of hydrogen and carbon dioxide to methane (hydrogenotrophic methanogenesis) and/or acetate to methane (acetoclastic methanogenesis). A single methanogenic MAG classified as *Methanobacterium* was recovered. *Methanobacterium* revealed genes for hydrogenotrophic methanogenesis in its proteome (**Figure 4**). The [NiFe] hydrogenases coenzyme-F_420_-reducing hydrogenase (EC:1.12.98.1) and coenzyme-F_420_-non-reducing hydrogenase (EC:1.12.99.-) were abundant in the proteome. The energy converting hydrogenases (*eha* and *ehb*) were also detected but were less abundant in the proteome. *Methanobacterium* also expressed the key enzymes for acetoclastic methanogenesis (ACSS, EC:6.2.1.1 and *cdh*, EC:2.1.1.245) (**Figure 4**). However, it has been reported that hydrogenotrophic methanogens may contain these key genes but use them for CO_2_ fixation and not acetate utilisation (Costa et al., 2013). *Methanobacterium* encoded all of the genes required for coenzyme M biosynthesis and F420 biosynthesis.

### Fermentative Bacteria

The MAGs *Clostridiales* and *Erysipelothrix* did not harbour any respiratory pathways and likely gain energy via fermentation. Both MAGs harboured a complete EMP pathway which was partially detected in their proteome, in addition to ABC transporters for the uptake of extracellular sugars and peptides. *Erysipelothrix* also encoded genes for the production of pyruvate via the Entner–Doudoroff (ED) pathway (**Figure 4**). Both MAGs encoded glucosidases for the hydrolysis of complex carbon compounds (EC:3.2.1.10, EC:3.2.1.21). The genome of *Clostridiales* encoded a number of peptidases and an almost complete pathway for lysine fermentation was found in the proteome indicating it can utilise peptides as a source of carbon and nitrogen. Both MAGs harboured the genes for acetate production from pyruvate and genes for the production of other small organic compounds (**Figure 4**). Ethanol and methanol were detected in the groundwater, consistent with alcohol fermentation. Furthermore, an alcohol dehydrogenase was detected in the genome of *Clostridiales* and *Hoeflea* (**Figure 4**), but not in the corresponding proteomes. Conversely, alcohol dehydrogenases were detected in the metaproteome, but they did not correspond to a MAG. The protein sequences were compared to the NCBI protein database and they shared the greatest sequence identity with alcohol dehydrogenases from *Acholeplasma* and *Sphaerochaeta*. *Acholeplasma* was abundant in 16S rRNA gene amplicon libraries (**Figure 2**) and hence may represent a genus that contributes to the production of alcohols. The genus *Sphaerochaeta* was not detected in 16S rRNA gene amplicon libraries but the corresponding class *Spirochaetales* was detected in low abundance (<0.01%).

### Sulphide Oxidising Bacteria

The sulphur oxidising protein *soxB* which forms part of the multi-enzyme Sox complex that oxidises reduced sulphur compounds (Meyer et al., 2007) was used as an indicator of sulphide oxidation. Microorganisms harbouring *soxB* represented a relatively minor proportion of the microbial community and SoxB peptides were not detected in the metaproteome (**Figure 3**). In anoxic environments, sulphide oxidation can be coupled to nitrate or nitrite reduction. Genes encoding the large subunits of nitrate reductases, *napA* and *narG* (EC:1.7.99.-), were sought as indicators of nitrate reduction to nitrite (**Figure 3**), the first step in dissimilatory nitrate reduction to ammonium (DNRA) and denitrification. The final step in the denitrification pathway produces dinitrogen gas and is catalysed by the enzyme nitrous oxide reductase, encoded by *nosZ* (EC:1.7.2.4). All three of these genes (*napA*, *narG*, and *nosZ*) were detected in the metagenome, suggesting the metabolic potential for nitrate metabolism but its activity was not supported by the detection of peptides from the corresponding proteins in the metaproteome (**Figure 3**).

Genes for the oxidation of sulphide were detected in *Hoeflea* and *Hydrogenophaga.* Both MAGs possessed the complete Sox system (*soxXYZABCD*) for the oxidation of thiosulphate, sulphite, sulphur and sulphide, in addition to the sulphide oxidising sulphide:quinone oxidoreductase (*sqr*, EC:1.8.5.4) gene. *Hoeflea* also encoded the sulphite oxidising enzyme *soeABC* (EC:1.8.5.6). The sulfane dehydrogenase subunit SoxC and Sqr were detected in the proteome of *Hoeflea*. No sulphide oxidising genes were detected in the proteome of *Hydrogenophaga*. However, because this genus was relatively low abundance, it is not surprising that few genes from *Hydrogenophaga* were detected in the metaproteome, which typically captures the more abundant proteins. *Hoeflea* was more abundant, but, nevertheless, relatively few genes were detected in its proteome. Therefore, it was not possible to fully elucidate which pathways were active in these microorganisms.

Both *Hoeflea* and *Hydrogenophaga* encoded the genes for carbon fixation via the reductive pentose phosphate pathway (**Figure 4**) and energy conservation by denitrification indicating that they have the potential to grow autotrophically, coupling sulphide oxidation to denitrification. Although sulphide is abundant in the groundwater, the electron acceptors nitrate and nitrite were not detected (**Supplementary Table S1**).

*Hydrogenophaga* additionally harboured seven [NiFe] hydrogenases from the Groups 1d, 2b, and 3d (**Figure 4**). Group 1d [NiFe] hydrogenases are respiratory and indicate hydrogen can be oxidised, while Group 2b are H_2_-sensing hydrogenases and control the expression of respiratory hydrogenases. Group 3d hydrogenases are bidirectional and can be used for either hydrogenotrophic carbon fixation, or fermentative production of hydrogen (Søndergaard et al., 2016).

## Discussion

**FIGURE 5:**
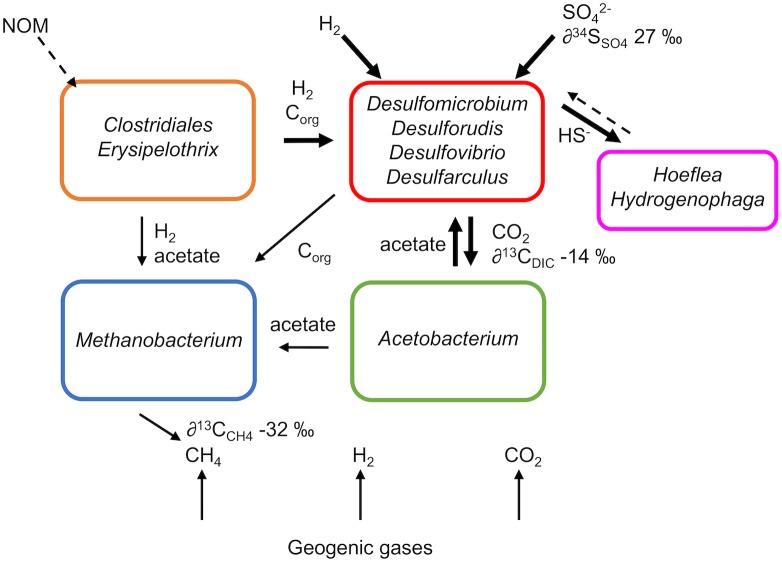
Metabolic model of microbial metabolism in subsurface groundwater from Olkiluoto inferred from metaproteogenomic data. NOM, natural organic matter. Dashed lines represent unconfirmed processes while solid lines represent confirmed processes. Line thickness is an indication of the predominance of the process.

The concentration of sulphide in methane-rich groundwater from a fracture at 530.6 mbsl is among the highest observed at Olkiluoto. The goal of this study is to uncover the electron donors fuelling sulphidogenesis and to explain the accumulation of sulphide in this system. Metaproteogenomics revealed active metabolisms within the microbial community (**Figures 3–5**). Unsurprisingly, SRB represented the most abundant metabolic group in the groundwater (**Figure 2**). SRB activity is corroborated through several lines of evidence. In addition to the accumulation of sulphide (1.3 ± 0.4 mM), the isotopic composition of sulphate (+27.26 ± 2.34‰) is consistent with sulphate reduction. The isotopic ratio shows an enrichment of ^34^S relative to other groundwaters at Olkiluoto with the same sulphate source (∼25‰; Posiva, 2013). This indicates that the lighter isotope (^32^S) has been preferentially removed by microbial activity. Furthermore, the presence of four MAGs pertaining to SRB and proteomic evidence for the full sulphate-reducing pathway in all MAGs provides irrefutable evidence of the activity of both *Deltaproteobacterial* and *Firmicutes* SRB.

More importantly, proteomic data indicated the presence of respiratory ([NiFe] Group 1) hydrogenases in all SRB MAGs, supporting oxidation of H_2_ as an electron donating process for sulphate reduction. Hydrogen was not detected in the groundwater, but it can be difficult to measure in environments where hydrogen-utilising microorganisms maintain the concentration at levels below detection (Pedersen, 2014). Hydrogen may be sourced from geogenic gases or as a metabolic product (**Figure 5**). Hydrogenases were also identified in the proteomes of *Acetobacterium* and *Methanobacterium* (**Figure 4**) suggesting hydrogen is an important electron donor for multiple taxa in this groundwater. This is consistent with previous models of a hydrogen driven subsurface biosphere (Kotelnikova and Pedersen, 1997; Pedersen, 1999).

Furthermore, genes that encode enzymes for the oxidation of small organic compounds including formate, acetate, ethanol and lactate were detected in SRB MAGs (**Figure 4**) suggesting that organic carbon as well as hydrogen is driving sulphidogenesis. Organic compounds generated by primary production or produced by fermentative microorganisms can provide an electron donor for SRB (**Figure 5**). In particular acetate (∼1.5 mM), acetone (∼8 μM), ethanol (∼29 μM) and methanol (∼65 μM) were detected in the groundwater (**Table 1**).

However, there must also be more complex organic substrates present to provide an energy source for fermentative microorganisms *Clostridiales* and *Erysipelothrix* (**Figure 5**). Detected organic compounds did not fully account for the total DOC (∼4.7 mM), suggesting that other organic compounds, not measured here, are also present. Diverse heterotrophic populations have also been reported elsewhere in the Fennoscandian Shield (Purkamo et al., 2015, 2016; Wu et al., 2016) despite complex organic carbon not typically being considered available in deep oligotrophic ecosystems with long residence times (>10,000 years), where photosynthesis-derived organic compounds from the surface are not replenished. The degradation of recalcitrant natural organic matter (NOM) offers one possible source of complex organic carbon in the deep subsurface. At Outokumpu, a site which also forms part of the Fennoscandian Shield in Finland, ancient organic carbon from the bedrock is proposed to supply heterotrophic microorganisms with organic carbon (Purkamo et al., 2015). Heterotrophic populations were also detected at Äspö, Sweden, where slow recharge from the Baltic Sea was considered to provide a source of organic carbon (Wu et al., 2016). Alternatively, the activity of bacteriophages detected in Olkiluoto groundwater may also provide a source of carbon (Pedersen, 2012) by inducing cell lysis and precipitating the associated release of small organic compounds and peptides into the carbon pool from cell necromass. Bacteriophages have been detected in groundwater from the Fennoscandian Shield (Kyle et al., 2008; Pedersen, 2013) and one lytic to *Pseudodesulfovibrio aespoeensis* has been isolated from groundwater at Äspö (Eydal et al., 2009). Furthermore, bacteriophage-induced cell lysis has been postulated to control numbers of microorganisms in subsurface Fennoscandian Shield microbial communities (Pedersen, 2012). Fermentative populations could degrade the released organic carbon and peptides, e.g., by the lysine fermentation pathway detected in the proteome of *Clostridiales*, and further produce small organic compounds that can be utilised by SRB.

δ^13^C_DIC_ values at Olkiluoto typically vary between −25‰ and −10‰, indicative of the degradation of organic carbon (Posiva, 2013). Due to the low DIC content in the methane-rich groundwater at Olkiluoto, there are few comparative δ^13^C_DIC_ values. One study of methane-rich, sulphate-depleted groundwater at Olkiluoto (863 m depth) reported a positive δ^13^C_DIC_ value (+16.8‰; Haveman and Pedersen, 2002). The positive δ^13^C_DIC_ value was consistent with methanogens and acetogens being the dominant metabolic groups resulting in a greater proportion of carbon dioxide consumption and removal of the lighter ^12^C. The δ^13^C_DIC_ values reported here (−14.19 to −16.93‰) indicate that carbon dioxide production (rather than consumption) is the dominant process contributing to the DIC pool (Posiva, 2013). This further supports the suggestion that SRB, the most abundant metabolic group, could contribute to the DIC pool by metabolising small organic compounds and releasing CO_2_. SRB MAGs expressed genes in the WL pathway (**Figure 4**) that can be used in the oxidative direction (oxidation of acetate to CO_2_) coupled to sulphate reduction (Ragsdale and Pierce, 2008) or in the reductive direction (CO_2_ fixation) coupled to biosynthesis. Thus, it is not possible to directly determine from the proteogenomic approach whether SRB may fix CO_2_. Nonetheless, it is thermodynamically unfavourable to fix CO_2_ in the presence of abundant acetate, suggesting that organic carbon oxidation is extant. The oxidation of other small organic compounds detected in the groundwater, such as ethanol, would also yield CO_2_.

Elevated sulphide concentrations have been previously reported in Olkiluoto where sulphate-rich and methane-rich groundwaters meet at 250–350 m depth (Pedersen, 2014; Bomberg et al., 2015). In those studies, increased cultivable numbers of SRB and the presence of archaea related to ANME implicated methane as one possible electron donor for sulphate reduction. However, there was insufficient evidence to support that methane contributed significantly to sulphidogenesis. At OL-KR46, sulphide, sulphate and methane are abundant in the groundwater, but there is no evidence that methane serves as an electron donor for sulphate reduction here. The isotopic signature of DIC precludes AOM as a significant metabolic process contributing to the DIC pool as the mineralisation of methane, which has a low δ^13^C_CH4_ value, should result in more negative δ^13^C_DIC_ values than observed here (Posiva, 2013; Sahlstedt et al., 2016). To date, only one sample from Olkiluoto has been found to have an isotopic signature consistent with the possible use of methane (or a higher hydrocarbon) as an electron donor (Posiva, 2013). Finally, the absence of AOM in OL-KR46 is also supported by the microbiological data as ANME archaea, which catalyse AOM, were not detected and all *mcrA* genes were related to the methanogenic *Methanobacterium*. If ANME archaea are present in the sulphate-rich groundwater that is drawn-down into the deeper methane-rich groundwater during mixing then they should still be detected, but they were not found in 16S rRNA gene amplicon libraries (**Supplementary Table S3**). It is possible that the low abundance (Pedersen, 2013; Pedersen et al., 2013), slow growing (Krüger et al., 2008; Nauhaus et al., 2005, 2007) ANME archaea were unable to establish in competition with faster growing SRB. In particular endospore-forming *Firmicutes*, which were abundant in OL-KR46 groundwater (**Figure 2**), are well-adapted to grow quickly in response to favourable environmental conditions and their frequent isolation from subsurface marine sediments has been attributed to their fast growth response (Parkes et al., 2014). In OL-KR46, the introduction of sulphate to deep groundwater containing electron donors for sulphate reduction (hydrogen and acetate) provides favourable environmental conditions for growth.

The repository at Olkiluoto will be at 400–450 m depth, below the natural sulphate-methane mixing zone. SRB are not usually abundant in the saline methane-rich groundwater (Haveman and Pedersen, 2002; Posiva, 2013). However, artificial mixing caused by disturbances from the construction of the repository could lead to SRB activity, as observed here, where the flow of the sulphate-rich groundwater to the methane-rich zone has resulted in an elevated concentration of sulphide. The introduction of sulphate-rich groundwater to deep methane-rich groundwater provides an electron acceptor in a system which is otherwise limited in terminal electron acceptors. Geogenic hydrogen and biogenic organic carbon (e.g., acetate) in the methane-rich groundwater offer both electron donor and carbon source facilitating SRB metabolism (**Figure 5**). Over the course of the 9-month sampling period, sulphate decreased by 0.3 mM (**Table 1**). It is possible that sulphate-reducing activity may be a relatively short-term issue if sulphate is depleted and not replenished by further down-flow of sulphate-rich groundwater. However, in this system, sulphate was depleted following the fracture being isolated by packers. Continuous mixing of sulphate-rich and methane-rich groundwater could occur during repository construction and throughout the unsaturated phase after repository closure, if water conducting fractures are disturbed. This could result in continued sulphidogenesis.

Sulphide can be removed from the system either by abiotic or biotic processes. Abiotically, ferrous iron (Fe^2+^) can react with sulphide (S^2-^) and precipitate as ferrous sulphide (FeS). However, sulphide is available in excess (∼1.5 mM) compared to Fe^2+^ in this system, as the latter was often below the limit of detection (<0.2 μM), enabling aqueous sulphide to persist. Alternatively, microorganisms can remove sulphide by oxidation. The MAGs *Hoeflea* and *Hydrogenophaga* harboured the metabolic potential for sulphide oxidation (**Figure 4**). Few proteins were detected from these MAGs; however, the moderate dynamic range achievable by metaproteomics usually reveals proteins from the more abundant organisms, and thus would miss expressed proteins from lower abundance organisms. The genes *soxC* and *sqr* were detected in low abundance in the proteome of *Hoeflea* suggesting it could be actively metabolising sulphide. The oxidation of sulphide could potentially mitigate sulphide-induced corrosion, whereby the sulphide produced by SRB is re-oxidised by sulphide oxidising bacteria. However, this strategy is only successful as long as there is a suitable electron acceptor for sulphide oxidation. Both *Hoeflea* and *Hydrogenophaga* genomes include the complete pathway for denitrification suggesting that they may couple sulphide oxidation to the reduction of nitrate. However, neither nitrate nor nitrite were detected in the groundwater (**Supplementary Table S1**) and peptides from the denitrification pathway were not detected in the metaproteome. MPN assays have detected nitrate-reducing microorganisms at Olkiluoto at multiple depths (0–600 m) despite nitrate always being very low or below detection limit (Posiva, 2013). Nitrate-reducing bacteria have also been detected at 500 m depth in groundwater from the Fennoscandian Shield at Outokumpu (Rajala et al., 2015). In that study, *narG* transcripts from *Epsilonproteobacteria* were detected in response to the addition of sulphate and methane, possibly as a result of the increased concentration of sulphide. However, *narG* transcripts were not detected in the unamended fracture water, suggesting either that these organisms were not actively reducing nitrate *in situ* or that they were low abundance. Nitrate-reducing bacteria could be inactive but remain viable and respond to substrate addition. However, a few proteins were detected from *Hoeflea* and *Hydrogenophaga* (e.g., sulphide oxidation Sox) suggesting that these organisms are active in OL-KR46 and that they are either utilising an alternative metabolism or there is an N source which is below detection limit. Our data presents insufficient direct evidence for active sulphide oxidation or an alternative metabolism for these two MAGs, but it does demonstrate the metabolic potential for nitrate-dependant sulphide oxidation.

## Conclusion

The metabolic model constructed from metaproteogenomic and isotopic data suggests that sulphidogenesis in methane-rich groundwater from drillhole OL-KR46 is fuelled by hydrogen and organic carbon (**Figure 5**). Despite the abundance of methane, there is no evidence to support that methane oxidation is a contributing metabolism. The low diversity community in OL-KR46 groundwater demonstrates the impact of the addition of sulphate to a system otherwise largely limited in electron acceptors. Carbon is cycled within the community of SRB, acetogens, methanogens and fermenters. Traditionally, the most energy yielding reactions (i.e., sulphate reduction) should outcompete the less thermodynamically favourable reactions (carbon dioxide reduction). However, this conventional view of redox zonation has been challenged, with evidence for methanogenesis and/or acetogenesis in the presence of sulphate and metal-reducing populations (Lever, 2012). Here, proteomic data suggest that SRB, methanogen and acetogen metabolic activity co-exist. While it was not possible to conclusively show nitrate-dependent sulphide oxidation, some evidence of this metabolism was uncovered and it remains a possible sink for sulphide. This work contributes to unravelling the microbial metabolic web in the deep granitic subsurface and provides conclusive findings about sulphate reduction in this system, which can be harnessed for nuclear waste disposal.

## Author Contributions

Field work and laboratory analyses were conducted by EB. MF assisted with the design and implementation of field work. GS and LB assisted with field work and geochemical laboratory analyses. JA and AA provided training and support for the analysis of metagenomic data. Metaproteomic analyses were performed by CQ and WX, supervised by RH. TL contributed to project conception and development. RB-L conceived and supervised the study. EB wrote the manuscript with contributions from all authors.

## Conflict of Interest Statement

The authors declare that the research was conducted in the absence of any commercial or financial relationships that could be construed as a potential conflict of interest.
