# Supplementary figures and images for: Biogeochemical Cycling by a Low-Diversity Microbial Community in Deep Groundwater
Source: Front Microbiol. 2018 Sep 7;9:2129. doi: 10.3389/fmicb.2018.02129 (PMC6137086; doi:10.3389/fmicb.2018.02129)

**Fig. S1:** Rarefaction curves on the species richness (Chao1; **A**) and diversity (PD whole tree; **B**).

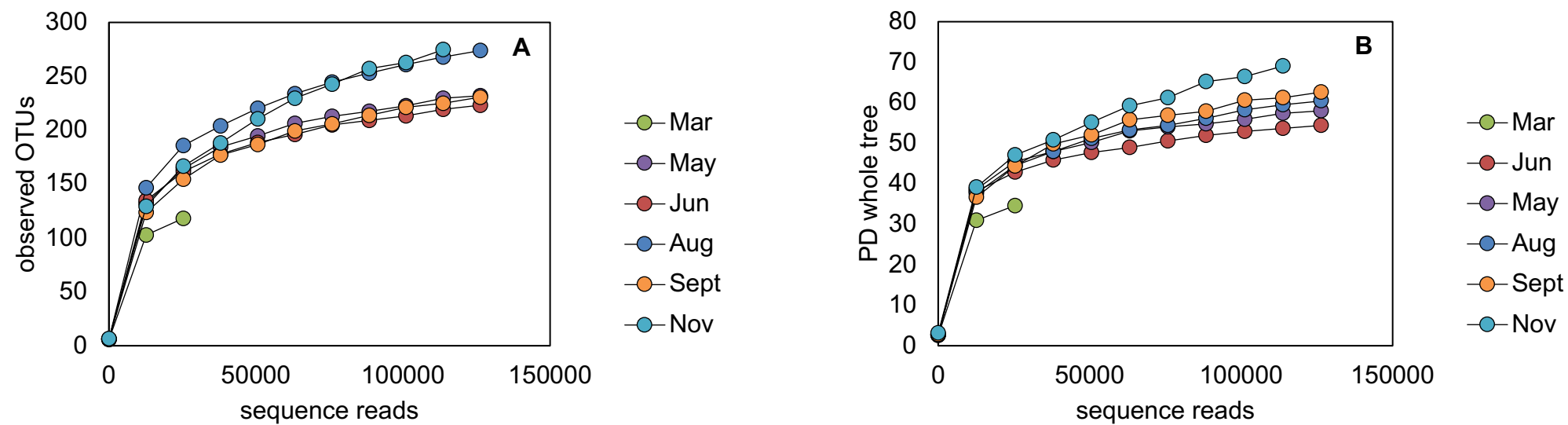

Supplement: Supplementary file 1 [file Data_Sheet_1.PDF]
